# Obesity-induced PARIS (ZNF746) accumulation in adipose progenitor cells leads to attenuated mitochondrial biogenesis and impaired adipogenesis

**DOI:** 10.1038/s41598-023-49996-0

**Published:** 2023-12-27

**Authors:** Kazuki Hachiya, Yusuke Deguchi, Takuro Hirata, Tomoya Arikawa, Hiroto Fukai, Tatsuhiro Esashi, Kota Nagasawa, Yuhei Mizunoe, Yuka Nozaki, Masaki Kobayashi, Yoshikazu Higami

**Affiliations:** 1 Laboratory of Molecular Pathology and Metabolic Disease, Faculty of Pharmaceutical Sciences, Tokyo University of Science, 2641 Yamazaki, Noda, 278-8510 Japan; 2 Department of Nutrition and Food Science, Graduate School of Humanities and Sciences, Ochanomizu University, Tokyo, Japan; 3 Institute for Human Life Innovation, Ochanomizu University, Tokyo, Japan; 4 Division of Cell Fate Regulation, Research Institute for Biomedical Sciences, Tokyo University of Science, 2669 Yamazaki, Noda, 278-8510 Japan

**Keywords:** Cell biology, Molecular biology

## Abstract

White adipose tissue (WAT) is critical for whole-body energy metabolism, and its dysfunction leads to various metabolic disorders. In recent years, many studies have suggested that impaired mitochondria may contribute to obesity-related decline in adipose tissue function, but the detailed mechanisms remain unclear. To investigate these mechanisms, we carried out a comprehensive analysis of WAT from mice with diet-induced obesity. We discovered the transcription factor Parkin interactive substrate (PARIS or ZNF746), which suppresses the expression of peroxisome proliferator-activated receptor γ coactivator-1α (PGC-1α), a key regulator of mitochondrial biogenesis, to be accumulated in adipose progenitor cells from obese mice. Furthermore, we demonstrated that 3T3-L1 preadipocytes with overexpression of PARIS protein exhibited decreased mitochondrial biogenesis and impaired adipogenesis. Our results suggest that the accumulation of PARIS protein may be a novel component in the pathogenesis of obesity-related dysfunction in WAT.

## Introduction

White adipose tissue (WAT) acts as an energy reservoir that maintains the energy balance of the entire body. Mature adipocytes, a key component of WAT, serve as the primary site for energy storage and adipokine secretion. Mature adipocytes store excess energy in the form of triglycerides and release stored energy when needed. Additionally, mature adipocytes secrete several types of bioactive molecules called adipokines, which influence energy metabolism throughout the body^1^. WAT also contains several other cell types, including adipose progenitor cells (APCs). APCs, the source of mature adipocytes, are considered a subpopulation of platelet-derived growth factor receptor alpha (PDGFRα)-positive mesenchymal stem cells (MSCs)^1,2^.

Recent studies have implicated impaired mitochondrial biogenesis in WAT in the development of obesity-associated pathologies^3,4^. In recent years, emerging evidence has underscored the role of peroxisome proliferator-activated receptor γ coactivator-1α (PGC-1α) as a key regulator of mitochondrial biogenesis in various tissues, including WAT^4,5^. Reportedly, the mitochondrial DNA (mtDNA) copy number and the expression of genes such as *Pgc-1α* and mitochondrial transcription factor A (*Tfam*), which are involved in mitochondrial biogenesis, are decreased in WAT of animals and humans with obesity^3,6^. Furthermore, downregulation or deletion of PGC-1α in WAT in genetically engineered mice has been associated with obesity-related whole-body metabolic abnormalities^4,7^. Therefore, reduced expression of *Pgc-1α* and subsequent attenuated mitochondrial biogenesis in WAT is speculated to be a major molecular event in the pathology of obesity. However, the mechanisms underlying the obesity-associated downregulation of *Pgc-1α* in WAT remain poorly understood.

Parkin interactive substrate (PARIS, also known as ZNF746), which was originally identified as a pathogenic substrate of the E3 ubiquitin ligase PARKIN in the brain of familial Parkinson's disease (PD) patients, reduces mitochondrial biogenesis by repressing *PGC-1α* transcription^8^. Under healthy conditions, PARIS protein levels are regulated by proteasomal degradation via phosphorylation by PINK1 and subsequent ubiquitination by PARKIN^9^. Abnormalities in this ubiquitin–proteasome system lead to nuclear accumulation of PARIS in the neurons of PD patients and PD model animals^9^. Previous studies have shown that nuclear accumulation of PARIS in neurons from PD patients and model animals suppresses mitochondrial biogenesis and promotes cell death^8,10,11^. Although reduced mitochondrial biogenesis is a critical event in the pathogenesis of many conditions in eukaryotes, the function of PARIS in other tissues and cells, including WAT, remains largely unknown.

To investigate the impact of PARIS in WAT with obesity, we analyzed gene and protein expression in WAT from diet-induced obese mice and in 3T3-L1 cells, a well-established preadipocyte cell line, undergoing adipogenesis. Our findings revealed that PARIS protein levels were increased in APCs in obese WAT, leading to mitochondrial dysregulation and impairment of adipogenesis. Our findings provide novel insights into the pathology of obese WAT.

## Results

### PARIS is present in large amounts in WAT

**Figure 1:**
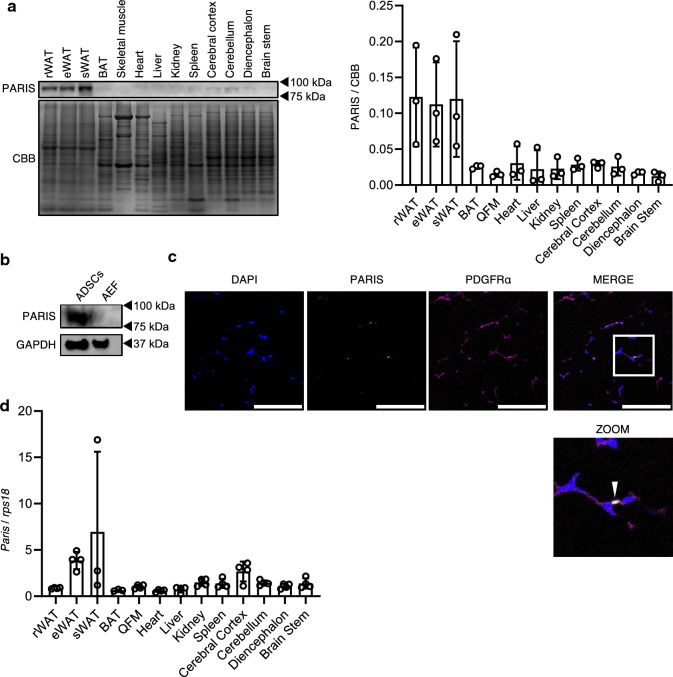
PARIS expression and localization analysis in healthy mice aged 20–25 weeks. (**a**) Western blot (left) and quantitative (right) analysis of PARIS protein levels in various mouse tissues (n = 3). (**b**) PARIS protein levels in ADSCs and the AEF. GAPDH was used as a loading control. (**c**) Fluorescent staining showing PARIS localization in the nucleus of PDGFRα-positive cells. Scale bars = 100 µm. (**d**) mRNA levels of *Paris* in several tissues (n = 3–4). Data are expressed as the mean ± standard deviation (SD). rWAT, retroperitoneal WAT; eWAT, epididymal WAT; sWAT, subcutaneous WAT; BAT, brown adipose tissue; QFM, quadriceps femoris muscle.

First, we examined PARIS protein levels in various tissues from wild-type healthy mice at 20–25 weeks of age and were surprised to find that WAT had higher levels than other tissues, including neural tissues, where PARIS is pathologically implicated (Fig. 1a). To examine the cellular distribution of PARIS in WAT, we compared its protein levels in the adipocyte-enriched fraction (AEF) and adipose tissue-derived stem cells (ADSCs) obtained from the stromal vascular fraction (SVF) of WAT. Interestingly, PARIS protein levels were significantly higher in ADSCs than in the AEF (Fig. 1b). Most ADSCs are APCs^12,13^. We confirmed that PARIS was localized in the nucleus of PDGFRα-positive progenitor cells in WAT in vivo using immunostaining (Fig. 1c). However, the pattern of *Paris* mRNA expression in the different tissues was not consistent with the pattern of protein expression in these tissues (Fig. 1d). These results suggested that the higher abundance of PARIS in ADSCs was likely due to regulation at the protein level rather than at the transcriptional level.

### Obesity increases PARIS protein levels in progenitor cells

**Figure 2:**
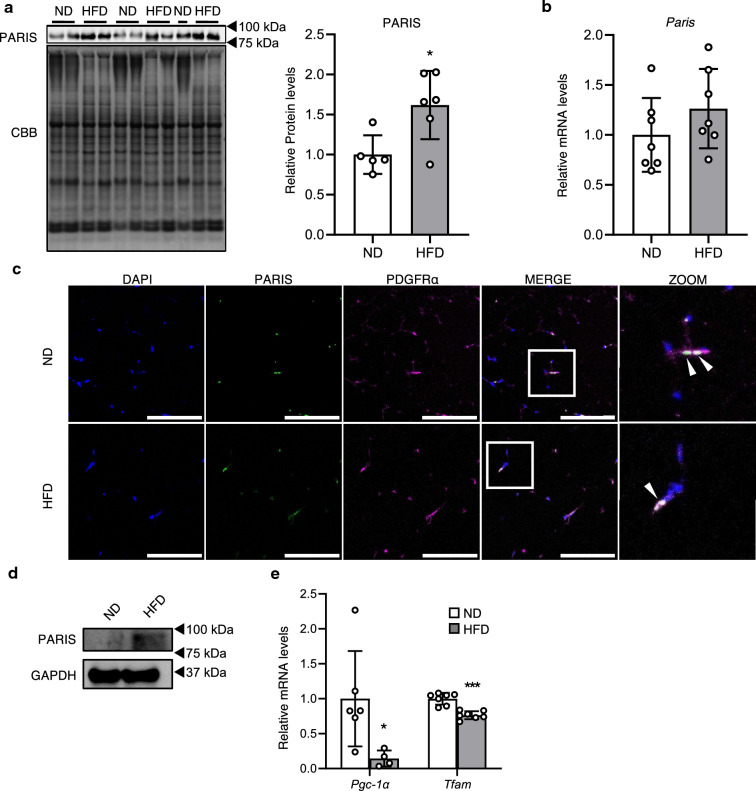
Analysis of PARIS quantity and localization in obese WAT. (**a**) Western blotting (left) and quantitative analysis (right) of PARIS protein levels in control ND and HFD mice (n = 5–6). (**b**) mRNA levels of *Paris* in WAT of ND and HFD mice (n = 7). (**c**) Fluorescent staining showing PARIS localization in the nucleus of PDGFRα-positive cells. Scale bars = 100 µm; ZOOM = magnification of the area indicated by the white rectangle, showing PARIS localization in the nucleus of PDGFRα-positive cells (arrowheads). (**d**) PARIS protein levels in ADSCs from ND and HFD mice (n = 5, pooled sample). GAPDH was used as a loading control. The blots of each protein were cropped from different parts of the same membrane. The original blots are presented in Supplementary Information 2. (**e**) mRNA levels of two regulators of mitochondrial biogenesis, *Pgc-1α* and *Tfam*, in ADSCs from HFD mice (n = 4–7). Data are expressed as the mean ± SD, and were analyzed by Student’s t-test; *p < 0.05, ***p < 0.001.

PARIS is degraded by the ubiquitin–proteasome system^9^. The function of the ubiquitin–proteasome system is upregulated in the early stage of adipogenesis in human ADSCs^14^, but is impaired in WAT of metabolically unhealthy patients with obesity^15^. Therefore, we hypothesized that PARIS degradation by proteasome activation occurs during the early stages of adipogenesis, and that PARIS accumulates in APCs in obese WAT. To test this hypothesis, we first compared PARIS protein levels in WAT isolated from high-fat diet-induced obese (HFD) mice and normal diet-fed (ND) mice. As expected, the protein levels of PARIS were higher in the WAT from HFD mice (Fig. 2a). However, the mRNA levels of *Paris* did not show a significant increase with obesity (Fig. 2b). Next, we used immunostaining of PARIS in WAT to demonstrate that PARIS was localized in the nucleus of PDGFRα-positive cells in both HFD and ND mice (Fig. 2c). We also observed that PARIS protein was more abundant in ADSCs derived from obese WAT (Fig. 2d). These results indicate that PARIS accumulates in ADSCs in obese WAT. PARIS inhibits mitochondrial biogenesis by suppressing the expression of *Pgc-1α* and *Tfam* in dopaminergic neurons^8,11^; therefore, we next analyzed the expression levels of these two genes in ADSCs from ND and HFD mice. Consistent with previous studies in dopaminergic neurons, both genes showed lower expression in ADSCs from HFD mice, which contained higher levels of PARIS than ND mice (Fig. 2e).

### PARIS levels decrease with adipogenesis

**Figure 3:**
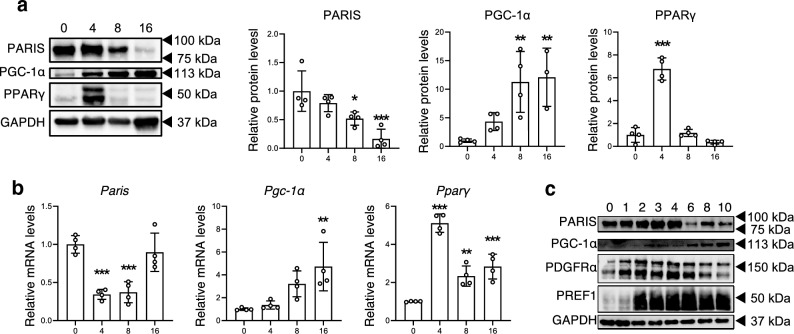
Western blotting and reverse transcription (RT)-PCR analysis of the indicated factors in 3T3-L1 cells and MEFs during adipogenesis. (**a**) Western blotting (left) and quantitative analysis (right) of the indicated proteins in 3T3-L1 cells induced to differentiate into white adipocytes and harvested at 0, 4, 8, and 16 days post induction. Successful adipogenesis was confirmed by increased protein levels of PPARγ. GAPDH was used as a loading control (n = 4). The blots of each protein were cropped from different parts of the same membrane. The original blots are presented in Supplementary Information 2. (**b**) RT-PCR analysis of *Paris*, *Pgc-1α*, and *Pparγ* mRNA levels in 3T3-L1 cells under the same conditions. (**c**) Western blotting of the indicated proteins from MEFs induced to differentiate into white adipocytes and harvested at 0, 1, 2, 3, 4, 6, 8, and 10 days post induction. The blots of each protein were cropped from different parts of the same membrane. The original blots are presented in Supplementary Information 2. Data are expressed as the mean ± SD and were analyzed by one–way ANOVA followed by Dunnett’s test; *p < 0.05, **p < 0.01, ***p < 0.001.

Our results indicated that PARIS is expressed in progenitor cells, but not in mature adipocytes in WAT. Furthermore, as mentioned above, we hypothesized that PARIS is degraded during adipogenesis. Therefore, we analyzed PARIS protein levels during adipogenesis in 3T3-L1 preadipocytes and found that PARIS levels decreased with adipogenesis (Fig. 3a). Successful adipogenesis was confirmed by changes in protein and mRNA levels of PPARγ in line with the trends described in previous reports^16,17^ (Fig. 3a, b). PARIS protein levels decreased with adipogenesis and, consistent with previous studies, both protein and mRNA levels of PGC-1α, which is known to be suppressed by PARIS, increased in parallel (Fig. 3a, b). These changes in protein levels of PARIS and PGC-1α during adipogenesis were confirmed using mouse embryonic fibroblasts (MEFs) (Fig. 3c). Consistent with the in vivo results in this study, the mRNA levels of *Paris* did not correlate with its protein levels during adipogenesis in 3T3-L1 cells (Fig. 3a, b) and MEFs (Fig. 3c).

Adipocyte differentiation can be broadly divided into two stages: (1) commitment by a subpopulation of PDGFRα-positive multipotent mesenchymal stem cells to become preadipocyte factor 1 (PREF-1)-positive APCs and: (2) differentiation of APCs into mature adipocytes^1^. Here, we demonstrate that, although PARIS levels decreased from day 6 during adipogenesis (Fig. 3c), there was no significant change in PARIS protein levels during the commitment from PDGFRα-positive fibroblasts to PREF1-positive preadipocytes on day 3 (Fig. 3c).

### Excessive PARIS levels inhibit mitochondrial biogenesis

**Figure 4:**
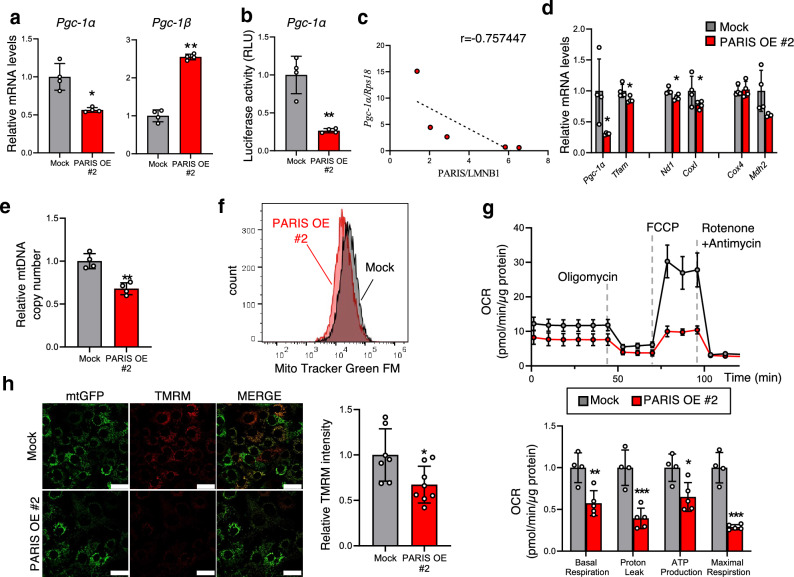
Evaluation of mitochondrial biogenesis and functions in PARIS-OE cells. (**a**) RT-PCR analysis of mRNA levels of *Pgc-1α* and *Pgc-1β* in 3T3-L1 cells undergoing mock transfection or transfection with a PARIS-OE vector (n = 4). (**b**) Luciferase reporter assay of *Pgc-1α* promoter activity in PARIS-OE and mock-transfected cells (n = 4). (**c**) Negative correlation between *Pgc-1α* mRNA levels and PARIS protein levels in PARIS-OE cells (n = 5). (**d**) RT-PCR analysis of mRNA levels of genes involved in mitochondrial biogenesis (*Pgc-1α* and *Tfam)*, and those that encode mitochondrial proteins on mtDNA (*Nd1* and *Cox1*) and nuclear DNA (*Cox4* and *Mdh2*) in PARIS-OE and mock-transfected cells (n = 4). (**e**) Decreased copy number of mtDNA in PARIS-OE cells, estimated by the fold change in expression of *CoxII* relative to that of *Hprt* (n = 4). (**f**) Mitochondrial mass analysis showing a decreased signal intensity in PARIS-OE cells. (**g**) Significant decrease in OCR in PARIS-OE cells (n = 4–5). Data are expressed as the mean ± SD. (**h**) Confocal microscopy (left) and quantitative analysis (right) showing reduced mitochondrial membrane potential in cells with mtGFP/FLAG-PARIS OE. Scale bars = 50 µm. Data obtained from 7–8 visual fields are expressed as the mean ± SD and were analyzed by Student’s t-test; *p < 0.05, **p < 0.01, *** p < 0.001. RLU, relative light units.

Because ADSCs from HFD mice produced greater amounts of PARIS protein than ADSCs from ND mice, we sought to mimic the conditions of ADSCs derived from obese WAT using a retroviral system to establish 3T3-L1 preadipocytes with PARIS overexpression (OE). We first showed that, compared with mock-transfected cells, both the expression levels of *Pgc-1α* and its promoter activity, as confirmed by luciferase assays, were decreased in PARIS-OE cells (Fig. 4a,b), and that the expression levels of *Pgc-1α* negatively correlated with the levels of PARIS OE (Fig. 4c). *Pgc-1β* also contributes to adipogenesis^18^ and in contrast to *Pgc-1α*, the expression levels of *Pgc-1β*, which is not a direct target of PARIS^19^, were increased in PARIS-OE cells (Fig. 4a). Additionally, PARIS OE cells exhibited decreases in the mRNA levels of mtDNA-encoded *Cox1* and *Nd1,* which produce mitochondrial proteins, the copy number of mtDNA, and the mass of mitochondria, indicating a decrease in mitochondrial biogenesis (Fig. 4d–f). By contrast, there were no significant differences in the mRNA levels of *Cox4* and *Mdh2*, which produce nuclear-encoded mitochondrial proteins, with and without PARIS OE. Furthermore, we observed a decrease in the oxygen consumption rate (OCR), which serves as an indicator of mitochondrial oxidative phosphorylation (OXPHOS), in PARIS-OE cells (Fig. 4g). A decreased OCR can indicate a decreased number of mitochondria or decreased function of each mitochondrion, or both; therefore, we examined the mitochondrial membrane potential to determine the underlying cause. This analysis revealed a decrease in the intensity of tetramethylrhodamine methyl ester (TMRM) fluorescence in PARIS-OE cells, indicating a reduction in the mitochondrial membrane potential (Fig. 4h). Overall, these results indicate a decline in both mitochondrial quality and quantity in PARIS-OE cells. A different clone of PARIS-OE cells also showed decreases in both mtDNA copy number and membrane potential (see Supplementary Fig. S1 online).

**Figure 5:**
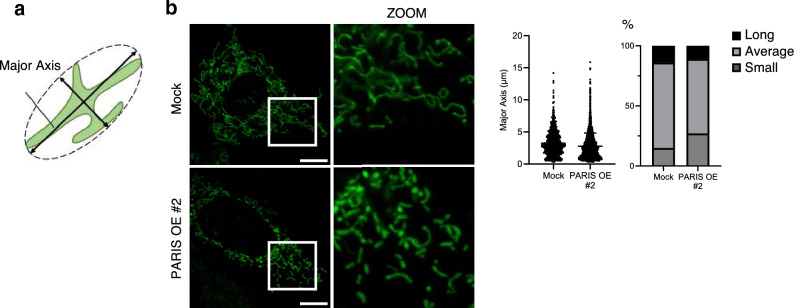
Confocal microscopy analysis of mitochondrial major axis under PARIS OE. (**a**) Schematic illustration of the mitochondrial major axis. (**b**) Representative images (left) and quantitative analysis (right) of 3T3-L1 cells undergoing mock transfection or transfection with a PARIS-OE vector. Scale bars = 10 µm; ZOOM = magnification of the area indicated by the white rectangle. Mitochondria were divided into three groups by size: Small = smaller than 1.29 µm; Average = between 1.29 and 5.19 µm; Long = longer than 5.19 µm. Notably, 1.29 µm is the value of the mean minus one standard deviation of the control, while 5.19 µm is the mean plus one standard deviation of the control.

Next, we analyzed mitochondrial size distribution under PARIS OE by measuring the mitochondrial major axis, as described by Rohani and colleagues^20^. A schematic illustration is shown in Fig. 5a, and representative images are shown in Fig. 5b. The proportion of smaller mitochondria was greater in PARIS-OE cells compared with that in mock-transfected cells (Fig. 5b). However, it should be noted that mitochondria longer than 15 µm were rarely observed in this experiment because of the inability of confocal microscopes to capture images beyond a certain depth.

### Excessive PARIS levels inhibit adipogenesis

**Figure 6:**
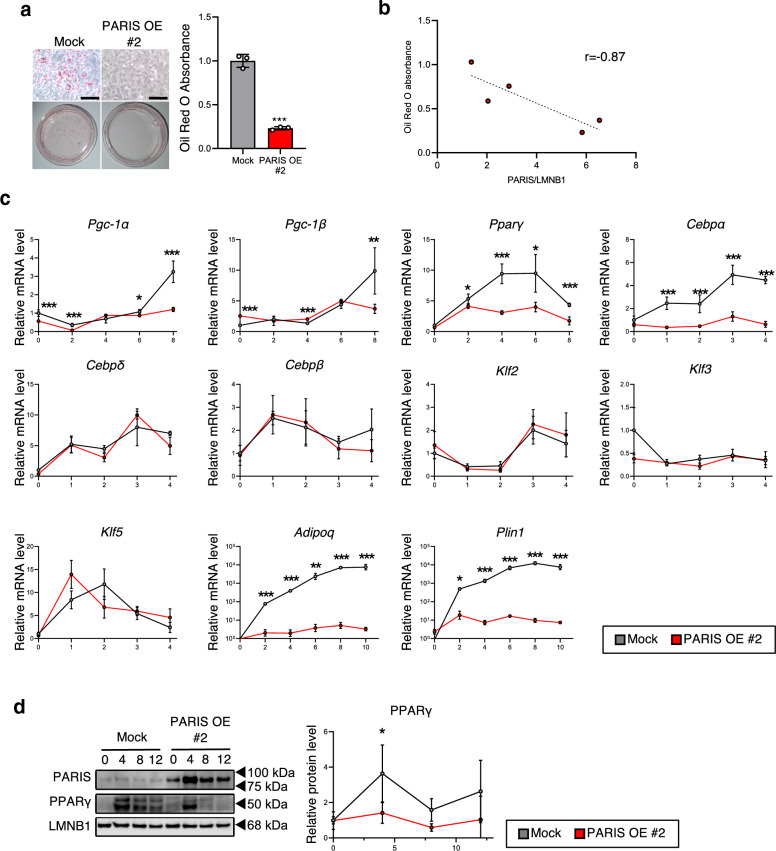
Analysis of adipocyte differentiation under PARIS-OE. (**a**) Oil red O staining of lipids (left) and quantitative analysis (right) of 3T3-L1 cells with mock transfection or PARIS-OE at 8 days post induction of differentiation (n = 3). Scale bars = 100 µm. (**b**) Scatter plot showing the negative correlation between PARIS protein levels and Oil red O absorbance in each clone at 8 days post induction of differentiation. (**c**) RT-PCR analysis of mRNA levels of eleven genes involved in different stages of adipocyte differentiation (n = 4). (**d**) Western blot (left) and quantitative analysis (right) of PPARγ expression in PARIS-OE cells at 0, 4 ,8, and 12 days post induction of differentiation (n = 4). Lamin B1 (LMNB1) was used as a loading control. The blots of each protein were cropped from different parts of the same membrane. The original blots are presented in Supplementary Information 2. Data are expressed as the mean ± SD and were analyzed by Student’s t-test; *p < 0.05, ***p < 0.001.

Knowing that mitochondrial biogenesis is required for adipogenesis^21^, we hypothesized that PARIS OE-induced suppression of mitochondrial biogenesis via suppression of *Pgc-1α* results in the impairment of adipogenesis. To test this hypothesis, we first analyzed the differentiation capacity of PARIS-OE cells. Oil red O staining showed that fewer lipid droplets accumulated in PARIS-OE cells compared with mock-transfected cells at 8 days post induction of differentiation (Fig. 6a). This decrease in lipid accumulation was negatively correlated with the level of PARIS OE (Fig. 6b; see Supplementary Figs. S1 and 2 online). Furthermore, the increase in mRNA levels of *Pgc-1α, Pgc-1β, Pparγ, Cebpα, Adipoq* and *Plin1* and the increase in protein levels of PPARγ were suppressed in PARIS-OE cells during adipogenesis (Fig. 6c,d). However, PARIS OE had no significant effect on the expression levels of *Cebpδ*, *Cebpβ*, *Klf2*, *Klf3,* and *Klf5*, which encode transcription factors that function upstream of *Pparγ* and *Cebpα* (Fig. 6c).

## Discussion

In this study, we found that progenitor cells in WAT contained relatively higher levels of PARIS than other tissues under normal conditions. Consistent with previous observations in dopaminergic neurons^19,22^, we discovered that PARIS decreased mitochondrial biogenesis and respiration and altered mitochondrial morphology in cultured APCs in vitro, with similar results in WAT. These findings suggest that the impact of PARIS extends across tissues. Importantly, we demonstrated that excessive PARIS levels decreased expression of mtDNA-encoded OXPHOS subunits, mitochondrial membrane potential, and OCR in vitro. APCs are a subpopulation of MSCs. Several studies have shown that OXPHOS is maintained at a low level in MSCs, whose metabolism is mainly dependent on glycolysis^23,24^. Additionally, many types of adult stem cells are known to have small and less polarized mitochondria, low levels of OXPHOS, and a primary dependence on glycolysis^25^. Döhla and colleagues reported that stem cells use the metabolic byproduct of glycolysis for biosynthetic reactions, specifically de novo purine synthesis, and that this metabolic state is required to maintain “stemness” in human stem-like cells^26^. Taken together, our results suggest that PARIS may play an important role in maintaining low respiration levels in APCs by decreasing mitochondrial biogenesis.

We also showed that PARIS protein is upregulated in APCs of obese WAT without an increase in the *Paris* mRNA level, suggesting a decreased rate of degradation by proteolytic systems, such as the ubiquitin–proteasome system or selective autophagy, among other potential mechanism. Diaz-Ruiz and colleagues reported that decreased proteasome activity led to accumulation of ubiquitinated proteins in obese WAT^15^. Therefore, we propose that PARIS protein accumulates in APCs under obese conditions, and that such accumulation could potentially be associated with dysfunction of the ubiquitin–proteasome system; however, further analysis is required to determine the precise degradation mechanism.

Notably, our data also suggest that the accumulation of PARIS protein in APCs may inhibit adipogenesis. Several studies have reported the impairment of APC differentiation in obese animals and humans^1,27^. This phenomenon can be interpreted in two different ways. We demonstrated here that white adipocyte differentiation is associated with an increase in PGC-1α. Furthermore, Ryu and colleagues reported that higher levels of OXPHOS are crucial for white adipocyte differentiation^28^, and they observed impaired adipogenesis when expression levels of mtDNA-encoded OXPHOS subunits were reduced. We found that cells with excessive amounts of PARIS exhibited impaired adipogenesis, lower levels of mtDNA-encoded OXPHOS subunits, lower OCRs, and decreased membrane potential. Taken together, these results suggest that accumulated PARIS protein in obese WAT impairs adipogenesis by suppressing OXPHOS. Alternatively, adipogenesis is known to be regulated by sequential activation of transcription factors^1^. Our in vitro study showed that the PARIS accumulated in APCs decreased adipogenesis by suppressing the expression of *Pparg* and *Cebpa*, without suppressing the expression of their upstream transcription factors. In support of these results, Yazar and colleagues reported that there is a PARIS binding motif in the promoter region of *Pparg* and that PARIS directly represses its expression in neurons of *Drosophila*^29^. Therefore, the impairment of differentiation in APCs with high levels of PARIS may be caused by the direct repression of *Pparg* expression by PARIS.

As discussed above, mitochondrial biogenesis through activation of *Pgc-1α* may contribute to adipogenesis. However, Ji and colleagues reported that mitochondrial biogenesis regulated by the activation of *Pgc-1β* is also essential for adipogenesis^18^. In this study, the increase in expression of both *Pgc-1α* and *Pgc-1β* during adipogenesis was suppressed in PARIS-OE cells, whereas expression of *Pgc-1α* decreased and *Pgc-1*β increased in PARIS-OE cells without adipogenic stimuli. These results suggest that, under basal conditions, the expression of *Pgc-1β*, which is not a direct target of PARIS^19^, increased in response to the suppression of *Pgc-1α* by PARIS in a compensatory manner. Taken together, a possible explanation is that although both *Pgc-1α* and *Pgc-1β* contribute to adipogenesis, the suppression of *Pgc-1α* by PARIS inhibits adipogenesis, and the suppressed increase in *Pgc-1β* expression is a consequence of impaired adipogenesis.

Contrary to our findings that suppression of *Pgc-1α* by PARIS inhibits adipogenesis, Pardo and colleagues reported that adipose-specific loss of *Pgc-1α* does not affect adipogenesis^30^. However, it is important to note that in their model, *Pgc-1α* is deleted along with the expression of *aP2*, a gene specific to mature adipocytes. In contrast, in our model, *Pgc-1α* is suppressed by PARIS in adipose progenitor cells. This indicates that the activation of *Pgc-1α* in progenitor cells, rather than in mature adipocytes, is crucial for adipogenesis. Furthermore, systemic *Pgc-1α* knockout mice have reduced weight gain in response to a high-fat diet^31^, indicating that the impaired adipogenesis may be linked to defects in mitochondrial biogenesis.

Overall, our findings suggest that accumulation of PARIS protein in APCs of obese WAT may inhibit adipogenesis through suppression of OXPHOS and/or direct repression of *PPARγ* expression. Several studies have shown that impaired adipogenesis in obese WAT leads to ectopic lipid accumulation, dyslipidemia, and insulin resistance^27^. Therefore, our findings strongly suggest that PARIS accumulation in APCs in WAT may be involved in the impairment of whole-body metabolism in individuals with obesity. In conclusion, our study provides new insights into the regulatory mechanism of adipogenesis in obesity and may contribute to the development of novel therapeutic strategies for obesity-related metabolic disorders.

## Methods

### Animals, isolation of ADSCs and collection of MEFs

The experimental procedures and reporting of this study were conducted in accordance with the ARRIVE guidelines. All animal experiments were conducted in accordance with ethical regulations for animal research and were approved by the Animal Experimentation Committees of Tokyo University of Science (Y21043, Y21045, Y22037). C57BL/6 mice were housed in a specific pathogen-free environment and had free access to food, a CRF-1 diet (Oriental Yeast, Tokyo, Japan), and water. At 8 weeks of age, mice were divided into two groups: one was fed a control CRF-1 diet (ND) and the other was fed High-Fat Diet 32 (CREA, Tokyo, Japan) (HFD). At 22 weeks of age, each group of mice was euthanized under isoflurane anesthesia (Wako, Osaka, Japan). The following tissues were removed, diced, frozen in liquid nitrogen, and stored at − 80 °C until use: retroperitoneal WAT (rWAT), epididymal WAT (eWAT), subcutaneous WAT (sWAT), brown adipose tissue (BAT), skeletal muscle, heart, liver, kidney, spleen, cerebral cortex, cerebellum, diencephalon, and brain stem.

ADSCs were isolated from the eWAT of each group of mice. The eWAT was dissected and digested using collagenase type II (Thermo Fisher Scientific, Waltham, MA, USA) at 37 °C for 30 min. Digested eWAT was filtered through a 100-µm cell strainer (BD Biosciences, Franklin Lakes, USA) and centrifuged at 300×*g* for 30 min at room temperature. The floating fraction was collected as the AEF, and the pellet was collected as the SVF. To isolate ADSCs from the SVF, the pellet was suspended with Dulbecco's Modified Eagle's Medium (DMEM; Wako) supplemented with 10% fetal bovine serum (FBS; Capricorn Scientific, Ebsdorfergrund, Germany) and 1% penicillin/streptomycin (Sigma‐Aldrich, St. Louis, MO, USA), transferred to a culture dish, and incubated in a cell culture chamber (37 °C, 5% CO_2_) until the cells reach 80–90% confluency. To remove floating cells in the SVF, cells were washed with phosphate-buffered saline (PBS), and the medium was exchanged daily.

MEFs from wild-type C57BL/6 mice were obtained by dissecting 13- to 15-day-old embryos (E13–15) from the uterus and washing with saline. The head, tail, limbs, and blood-enriched organs were removed, and the remaining tissues were washed with saline, minced, and trypsinized at 37 °C for 10 min. After inactivating the trypsin with FBS, MEFs were separated by filtration through a strainer, then cultured and passaged in maintenance medium comprising high‐glucose DMEM (Wako) supplemented with 10% FBS, 1% penicillin/streptomycin, and 0.1 µM 2-mercaptoethanol (2-ME; Wako). To induce differentiation, MEFs were cultured to confluence, at which time the medium was changed to differentiation medium consisting of maintenance medium containing 500 µM 3‐isobutyl‐1‐methylxanthine (Sigma‐Aldrich), 1 µM dexamethasone (Sigma‐Aldrich), 10 µg/mL insulin (Sigma‐Aldrich), and 100 µM troglitazone (Wako). The differentiation medium was changed every 2 days.

### Cell culture and differentiation

3T3‐L1 preadipocytes, purchased from RIKEN Bioresource Center (Ibaraki, Japan), were cultured in maintenance medium comprising DMEM supplemented with 10% FBS and 1% penicillin/streptomycin. For differentiation into mature adipocytes, the 3T3-L1 cells were cultured to confluence in maintenance medium, at which time the medium was changed to adipocyte differentiation medium (maintenance medium supplemented with 500 µM 3‐isobutyl‐1‐methylxanthine and 1 μM dexamethasone), and the cells were cultured for another 2 days. The adipocyte differentiation medium was then changed to adipocyte maturation medium (maintenance medium supplemented with 10 µg mL^−1^ insulin and 50 nM tri-iodothyronine (Sigma‐Aldrich), which was exchanged at 1‐day intervals. For this study, 3T3‐L1 adipocytes were differentiated for 16 days.

### Western blotting

Dissected tissues were lysed in lysis buffer (50 mM Tris–HCl pH 6.8, 2% sodium dodecyl sulfate [SDS], 3 M urea, 6% glycerol), sonicated, and boiled for 5 min. Protein concentrations of the soluble fraction were determined by the bicinchoninic acid protein assay (Thermo Fisher Scientific) following the manufacturer’s protocol, and standardized by the addition of lysis buffer. Protein samples were then mixed with 2-ME and bromophenol blue to obtain final concentrations of 5% and 0.025%, respectively, and boiled for 5 min. Lysates containing 15 µg protein were subjected to SDS–polyacrylamide gel electrophoresis, and the separated proteins were transferred to nitrocellulose membranes. The membranes were blocked with 2.5% skimmed milk and 0.25% bovine serum albumin (BSA) in Tris-buffered saline (50 mM Tris–HCl pH 7.4 and 150 mM NaCl) containing 0.1% Tween 20 (TBS-T) for 60 min at room temperature and then incubated with appropriate primary antibodies overnight at 4 °C. The membranes were then incubated with an appropriate secondary antibody (horseradish peroxidase-conjugated F(ab’)2 fragment of goat anti-mouse IgG or anti-rabbit IgG; Jackson ImmunoResearch, West Grove, PA, USA) for 60 min at room temperature. Finally, the membranes were incubated with ImmunoStar LD (Wako). Specific protein bands were visualized using ChemiDoc (Bio-Rad, Hercules, CA, USA), and the data were analyzed using Image Lab software.

### Image processing of original blots

The original blots are presented in Supplementary Information 2. Original images of full-length membranes cannot be included because the membranes were cut prior to hybridization with antibodies; therefore, all replicates performed for some blots are listed.

### RT-PCR

RNA was extracted from tissues and cell pellets using ISOGENII (Nippon Gene, Tokyo, Japan). Purified RNA was reverse transcribed using ReverTra Ace® qPCR RT Master Mix (Toyobo, Osaka, Japan) and cDNAs were amplified using a CFX Connect™ Real-time System in Thunderbird SYBR qPCR mix (Toyobo) with the primers for each gene, in accordance with the manufacturers’ protocols. Target gene expression data were normalized to *Rps18* expression.

### Immunostaining of WAT

Tissues were fixed by immersion in 10% neutral buffered formalin for over 24 h. Formalin-fixed paraffin-embedded tissue blocks were sliced into 5-µm thick sections. Tissue sections were deparaffinized with xylene and rehydrated with ethanol. Antigen retrieval was performed by treatment with 20 µg/mL proteinase K in PBS for 10 min. After permeabilization with PBS containing 0.1% (v/v) Triton X-100 (Sigma‐Aldrich), the sections were blocked with 1% BSA (Wako) in TBS-T for 60 min at room temperature. The sections were incubated overnight with appropriate primary antibodies at 4 °C. Thereafter, they were incubated with an appropriate secondary antibody and 4ʹ,6-diamidino-2-phenylindole (DAPI) stain. Stained tissues were visualized by high-resolution confocal microscopy (SP8; Leica, Wetzlar, Germany).

### Construction of plasmids for *Paris* overexpression and mitochondrial targeting sequence-fused green fluorescent protein (mtGFP) expression

To construct a retroviral plasmid for *Paris* overexpression, the coding region of *Paris* was amplified from eWAT cDNA using KOD FX Neo (Toyobo) and the following primers: 5′-TTT GAA TTC GCC ACC ATG GCC GAG GCG GCC GCG GC-3′ and 5′-TTT CTC GAG TTA CAG GTC TCC ACC ACC AT-3′. This amplicon was digested with EcoRI and XhoI, and inserted into the pMXs-AMNN-Puro plasmid^32^. The sequence of the recombinant *Paris* cDNA was confirmed to be identical to the mouse reference sequence (NM_001347142), and the OE plasmid was named pMXs-AMNN-Paris-Puro. The cDNA encoding DYKDDDDK (FLAG)-tagged PARIS was amplified by PCR using pMXs-AMNN-Paris-Puro and the following primers, and inserted into pMXs-AMNN-Puro plasmid as described above: 5′-TTT GAA TTC ACC ATG GAT TAC AAG GAT GAC GAC GAT AAG ATG GCC GAG GCG GCC GCG-3′ and 5′-TTT CTC GAG TTA CAG GTC TCC ACC ACC AT-3′. This plasmid was named pMXs-AMNN-FLAG-Paris-Puro. To construct the retroviral plasmid containing mtGFP, a fragment of the amino-terminal region of the precursor of cytochrome c oxidase subunit 8A was amplified from 3T3-L1 cDNA using PrimeSTAR HS DNA Polymerase (Takara, Shiga, Japan) and the following primers: 5′-TTT GGA TCC GTC ATG TCT GTC CTG ACG-3′ and 5′-TTT GAA TTC CCC TTC GAG TGG ACC TGA GC-3′. This amplicon was digested with BamH1 and EcoRI. The cDNA encoding GFP was obtained from CS-CDF-CD-PRE (RDB04379, RIKEN BRC DNA bank) using PrimeSTAR HS DNA Polymerase (Takara) and the following primers: 5′-TTT GAA TTC ACC GGT CGC CAC CAT GGT GAG CAA GGG CGA GG-3′ and 5′-TTT CTC GAG CTT ACT TGT ACA GCT CGT CC-3′. The fragments of the mitochondrial targeting signal and GFP cDNA were digested with BamH1 and EcoRI, and EcoRI and XhoI, respectively, and simultaneously subcloned into BamH1- and XhoI-digested pBluescript II SK(+). These fragments were subsequently cloned into the pMXs- Neo plasmid (kindly provided by T. Kitamura, The University of Tokyo, Japan) digested with the same restriction enzymes and named pMXs-mtGFP-Neo.

### Generation of PARIS-OE cells and mtGFP cells

PARIS-OE 3T3-L1 cells were generated using retroviral vectors, as reported previously^32^. Briefly, pMXs-AMNN-puro (Mock control), pMXs-AMNN-Paris-puro, or pMXs-AMNN-FLAG-Paris-Puro plasmids were transfected into Plat-E cells (kindly provided by T. Kitamura) using the calcium phosphate method. After 2 days, the supernatant from each virus-containing culture was harvested and filtered through a 0.22-µm filter (Millipore, Billerica, MA, USA). To establish PARIS-OE cells, 3T3-L1 cells were incubated in virus-containing medium for 2 days, followed by selection with 2 µg/mL puromycin for 5–7 days. To generate mtGFP cells, Mock and PARIS-OE cells were subjected to the same process using pMXs-mtGFP-Neo, and subsequently selected with 1 mg/mL G418 (Cayman Chemical, Ann Arbor, MI, USA) for 5–7 days.

### Oil Red O staining of 3T3-L1 cells

Formalin-fixed cells were stained with freshly prepared Oil Red O (Sigma‐Aldrich) staining solution (1.8 mg/mL in 60% isopropanol) for 20 min at room temperature. Cells were washed with 60% isopropanol and H_2_O. The stained cells were visualized using an optical microscope (BZ-9000; Keyence, Osaka, Japan). After visualization, the stained cells were incubated in isopropanol for 1 h at room temperature to dissolve the oil red O. The absorbance of oil red O in the lysate was then measured at 490 nm using a luminometer (EnVision; PerkinElmer, Waltham, MA, USA).

### Immunostaining of cells

Cells were fixed with 4% paraformaldehyde for 15 min and permeabilized with PBS containing 0.1% (v/v) Triton X-100 for 10 min. The cells were blocked with 5% goat serum and 1% BSA (Wako) in TBS-T for 60 min, incubated with appropriate primary antibodies overnight at 4 °C, and then incubated with an appropriate secondary antibody and DAPI. Stained cells were visualized by high-resolution confocal microscopy (SP8; Leica).

### Luciferase assay

Cells were seeded in 96-well plates at a density of 2.5–3.5 × 10^3^ cells/well. After 24 h, cells were co-transfected with a *PGC-1α* promoter firefly luciferase reporter plasmid and pGL4.74, a *Renilla* luciferase control plasmid for normalization, using TransIT®-2020 Transfection Reagent (Takara) in accordance with the manufacturer’s instructions. To construct the *PGC-1α* promoter reporter plasmid, we extracted the *PGC-1α* promoter region from *PGC-1α* promoter 2 kb luciferase (#8887, Addgene, Cambridge, MA, USA) using KpnI and XhoI, and then inserted it into the pGL4.10 vector. After transfection, cells were incubated for 24–72 h, and washed with PBS. Luciferase activity was measured using the Dual-Luciferase® Reporter Assay System (Promega, Madison, WI, USA) in accordance with the manufacturer’s protocol. Luminescence was recorded using a luminometer (EnVision; PerkinElmer). Relative luciferase activity was calculated by normalizing the firefly luciferase activity to the *Renilla* luciferase activity and expressed as a fold change compared with control samples.

### Extraction and quantification of mtDNA copy number

Extraction of total DNA, including mtDNA, was performed as previously described^33^. The copy number of mtDNA was estimated by the fold change in mtDNA-encoded *CoxII* (cytochrome c oxidase subunit II) expression relative to that of *Hprt* (hypoxanthine guanine phosphoribosyl transferase), which is encoded by genomic DNA, in total DNA.

### Mitochondrial mass analysis with fluorescence-activated cell sorting

Cells were treated with 200 nM Mito Tracker Green (Thermo Fisher Scientific) for 30 min, washed twice with PBS, and then detached using accutase (Nacalai Tesque, Kyoto, Japan). Next, the cells were passed through a 40-µm cell strainer (BD Biosciences) and resuspended in 2% FBS. Cells were analyzed with a flow cytometer (BD FACSMelody; BD Biosciences).

### Quantification of mitochondrial membrane potential

Cells plated on a glass-bottom dish (AGC Techno Glass Inc., Shizuoka, Japan) were treated with 25 nM TMRM, which reflects mitochondrial membrane potential, for 30 min. The cells were then washed three times with PBS and visualized by high-resolution confocal microscopy (SP8; Leica). The intensity of TMRM was quantified using ImageJ software.

### OCR measurement

OCR was measured using a Seahorse XF analyzer (Agilent Technologies, Santa Clara, CA, USA) with Agilent Seahorse XF/XF DMEM Medium, pH 7.4, containing 2 mM glutamine, 10 mM d-glucose, and 1 mM pyruvate. Measurements were performed in accordance with the manufacturer’s protocol. During the measurement, modulators of cellular respiration were added in the following order: 1.0 µM oligomycin, 0.5 µM cyanide-4-(trifluoromethoxy) phenylhydrazone (FCCP), and 5.0 µM rotenone + antimycin.

### Image analysis of mitochondria

The visualization of mitochondria was performed using a high-resolution confocal microscope (SP8; Leica). Images were analyzed using ImageJ software. The smallest ellipse surrounding each mitochondrion was assumed and its long diameter was quantified as the major axis. Mitochondria longer than 15 µm were rarely observed.

### Antibodies

Antibodies against the following proteins were used in this study: GAPDH (Santa Cruz, Dallas, TX, USA), LAMIN B1 (MBL, Tokyo, Japan), PARIS (Millipore), PARIS (Proteintech, Chicago, IL, USA), PGC-1α (Millipore), PDGFRα (R&D, Minneapolis, MN, USA), PPARγ (Santa Cruz) HRP-conjugated F(ab’)2 fragment of goat anti-rabbit IgG (Jackson ImmunoResearch), HRP-conjugated F(ab’)2 fragment of goat anti-mouse IgG (Jackson ImmunoResearch), HRP-conjugated F(ab’)2 fragment of donkey anti-goat IgG (Jackson ImmunoResearch), Alexa Fluor® 594 F(ab’)2 fragment of goat anti-mouse IgG (Invitrogen, Waltham, MA, USA), Alexa Fluor™ 488 F(ab’)2 fragment of goat anti-mouse IgG (H + L) (Invitrogen), and Alexa Fluor™ 594 F(ab’)2 fragment of goat anti-rabbit IgG (H + L) (Invitrogen).

### PCR primers

The following primer pairs were used for PCR amplification of target genes:

*Cebpa*: forward (F), 5′-GCCTTCAACGACGAGTTCC-3′; reverse (R), 5′-CCCGGGTAGTCAAAGTCACC-3′

*Cebpb*: F, 5′-AAGATGTTCCTGCGGGGTTG-3′; R, 5′-CACTTTAATGCTCGAAACGGAAAAG-3′

*Cebpd*: F, 5′-GTTCATTCTCTCCCGCACAC-3′; R, 5′-GAAACGCGTCCATCTCCTTAC-3′

*Cox I*: F, 5′-TGGTGGTCTAACCGGAATTG-3′; R, 5′-TGGGCTTTTGCTCATGTGTC-3′

*Cox IV*: F, 5′-CATTTCTACTTCGGTGTGCCTTC-3′; R, 5′-CACATCAGGCAAGGGGTAGTC-3′

*Klf2*: F, 5′-ACCAAGAGCTCGCACC-3′; R, 5′-TCCTTCCCAGTTGCAA-3′

*Klf3*: F, 5′-TACAGGAGAAAAGCCGTACAAATG-3′; R, 5′-TCATCAGACCGAGCGAACTTC-3′

*Klf5*: F, 5′-CCGGAGAGACGATCTGAAACAC-3′; R, 5′-GGAGCTGAGGGGTCAGATACTT-3′

*Mdh2*: F, 5′-AGGTTGACTTTCCCCAAGACC-3′; R, 5′-CATAAGCCATGGACAGAGTGG-3′

*Nd1*: F, 5′-CATAAGCCATGGACAGAGTGG-3′; R, 5′-ACGGAAGCCGTGGATAAGATG-3′

*Paris*: F, 5′-GGAGAATCTGCTACGCAACC-3′; R, 5′-GATGGCATAGTCCAGGGAGA-3′

*Pgc-1α*: F, 5′-AGACGGATTGCCCTCATTTG-3′; R, 5′-CAGGGTTTGTTCTGATCCTGTG-3′

*Pparg*: F, 5′-CACAATGCCATCAGGTTTGG-3′; R, 5′-GCGGGAAGGACTTTATGTATGAG-3′

*Rps18*: F, 5′-TGCGAGTACTCAACACCAACAT-3′; R, 5′-CTTTCCTCAACACCACATGAGC-3′

*Tfam*: F, 5′-CGGCTCAGGGAAAATTGAAG-3′; R, 5′-TCCAACTTCAGCCATCTGCTC-3′

*Cox II*: F, 5′-CCATCCCAGGCCGACTAA-3′; R, 5′-AATTTCAGAGCATTGGCCATAGA-3′

*Hprt1*: F, 5′-TGGGAGGCCATCACA-3′; R, 5′-TCCAGCAGGTCAGCAA-3′

### Statistical analysis

Statistical analyses were performed using GraphPad Prism. Student’s t-test and one-way ANOVA, followed by Dunnett’s test, were applied for between-group comparisons. Differences with *p* values < 0.05 were considered statistically significant.

### Supplementary Information

Supplementary Figures.

Supplementary Information 2.

## Data Availability

The data that support the findings of this study are available from the corresponding author upon reasonable request. The data are not publicly available due to privacy or ethical restrictions.
