# Supplementary material for: Obesity-induced PARIS (ZNF746) accumulation in adipose progenitor cells leads to attenuated mitochondrial biogenesis and impaired adipogenesis
Source: Sci Rep. 2023 Dec 27;13:22990. doi: 10.1038/s41598-023-49996-0 (PMC10752882; doi:10.1038/s41598-023-49996-0)
Supplement: Supplementary file 1 — Supplementary Figures. [file 41598_2023_49996_MOESM1_ESM.pptx]

## Slide 1
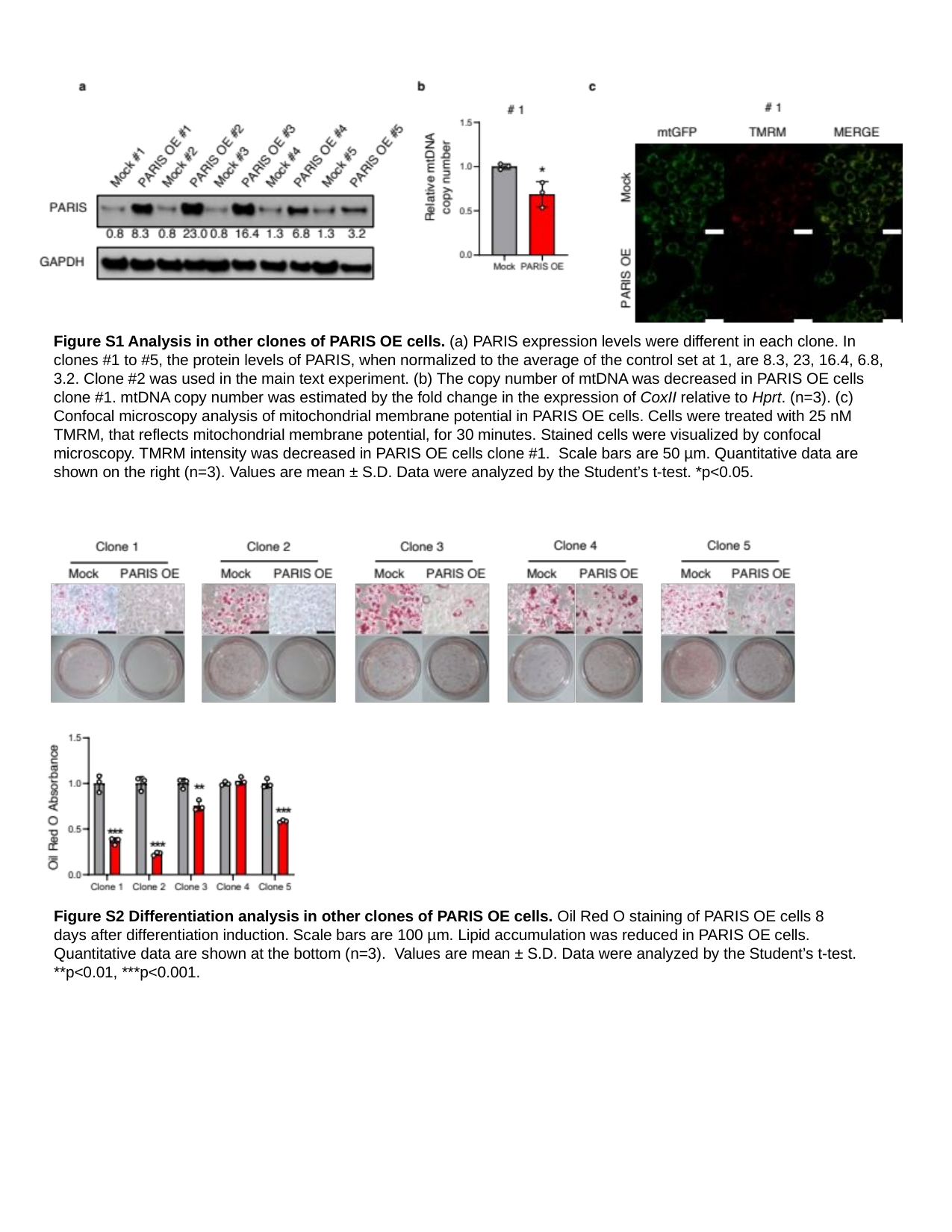

Figure S1 Analysis in other clones of PARIS OE cells. (a) PARIS expression levels were different in each clone. In clones #1 to #5, the protein levels of PARIS, when normalized to the average of the control set at 1, are 8.3, 23, 16.4, 6.8, 3.2. Clone #2 was used in the main text experiment. (b) The copy number of mtDNA was decreased in PARIS OE cells clone #1. mtDNA copy number was estimated by the fold change in the expression of CoxII relative to Hprt. (n=3). (c) Confocal microscopy analysis of mitochondrial membrane potential in PARIS OE cells. Cells were treated with 25 nM TMRM, that reflects mitochondrial membrane potential, for 30 minutes. Stained cells were visualized by confocal microscopy. TMRM intensity was decreased in PARIS OE cells clone #1. Scale bars are 50 µm. Quantitative data are shown on the right (n=3). Values are mean ± S.D. Data were analyzed by the Student’s t-test. *p<0.05.
Figure S2 Differentiation analysis in other clones of PARIS OE cells. Oil Red O staining of PARIS OE cells 8 days after differentiation induction. Scale bars are 100 µm. Lipid accumulation was reduced in PARIS OE cells. Quantitative data are shown at the bottom (n=3). Values are mean ± S.D. Data were analyzed by the Student’s t-test. **p<0.01, ***p<0.001.
